# Microenvironment in Oral Potentially Malignant Disorders: Multi-Dimensional Characteristics and Mechanisms of Carcinogenesis

**DOI:** 10.3390/ijms23168940

**Published:** 2022-08-11

**Authors:** Shuzhi Deng, Shimeng Wang, Xueke Shi, Hongmei Zhou

**Affiliations:** State Key Laboratory of Oral Diseases, Frontier Innovation Center for Dental Medicine Plus, West China School of Stomatology, Sichuan University, Chengdu 610041, China

**Keywords:** cancer chemoprevention, carcinogenesis, immune–metabolic–mechanical microenvironment, oral potentially malignant disorders

## Abstract

Oral potentially malignant disorders (OPMDs) are a group of diseases involving the oral mucosa and that have a risk of carcinogenesis. The microenvironment is closely related to carcinogenesis and cancer progression by regulating the immune response, cell metabolic activities, and mechanical characteristics. Meanwhile, there are extensive interactions between the microenvironments that remodel and provide favorable conditions for cancer initiation. However, the changes, exact roles, and interactions of microenvironments during the carcinogenesis of OPMDs have not been fully elucidated. Here, we present an updated landscape of the microenvironments in OPMDs, emphasizing the changes in the immune microenvironment, metabolic microenvironment, mechanical microenvironment, and neural microenvironment during carcinogenesis and their carcinogenic mechanisms. We then propose an immuno–metabolic–mechanical–neural interaction network to describe their close relationships. Lastly, we summarize the therapeutic strategies for targeting microenvironments, and provide an outlook on future research directions and clinical applications. This review depicts a vivid microenvironment landscape and sheds light on new strategies to prevent the carcinogenesis of OPMDs.

## 1. Introduction

OPMDs refer to any oral mucosal abnormality associated with an increased risk of developing oral squamous cell carcinoma (OSCC) [1]. They mainly include oral leukoplakia (OLK), oral lichen planus (OLP), oral submucous fibrosis (OSF), oral lichenoid lesions (OLL), and oral erythroleukoplakia (OEL) [1]. The overall rate of carcinogenesis across all OPMDs types is 7.9% [2]. The rate of carcinogenesis in the individual OPMDs is 1.4% for OLP, 9.5% for OLK, 3.8% for OLL, 5.2% for OSF, and 33.1% for OE [2]. The 5 year overall survival rate of OSCC patients is still less than 60%, which affects the patient’s quality of life and mental health greatly [3].

The microenvironment of OPMDs means the cells, molecules, and structures (such as blood vessels) that surround and support the cells and tissue of OPMDs. It undergoes changes and promotes the carcinogenesis of OPMDs significantly [4,5,6,7]. The potentially malignant cells, which refers to the cells that have a potential for carcinogenesis, undergo metabolic reprogramming. It usually inhibits the activation and function of immune cells, and remodels the extracellular matrix (ECM) [4,6]. The immune cells also undergo metabolic reprogramming and remodel the ECM to adapt to adverse conditions [8,9]. Vice versa, the changes in the ECM impede the immune cell response and change potentially malignant cells’ metabolic behaviors [10,11]. Meanwhile, the nervous system plays a regulatory role in these microenvironments [7]. Furthermore, there are extensive and profound interactions among microenvironments, which amplify the carcinogenic effects [12,13]. Therefore, it is significant to elucidate the dynamic changes in microenvironments and look for targets for cancer chemoprevention.

Several recent studies focused on the changes of some microenvironmental components during OPMDs carcinogenesis and the possible carcinogenic mechanisms. However, there is still a lack of a comprehensive review on the changes of various microenvironments during OPMDs carcinogenesis, their roles in carcinogenesis, interactions among microenvironments, and prevention or treatment strategies to target microenvironments. In this review, we mainly focus on the dynamics of the microenvironments during OPMDs carcinogenesis, and emphasis their possible roles in cancer initiation. We then illustrate the interactions among the immuno–metabolic–mechanical–neural environments. Finally, we provide an outlook on regulating microenvironments, to, thus, promote cancer chemoprevention.

## 2. Immune Microenvironment

**Figure 1:**
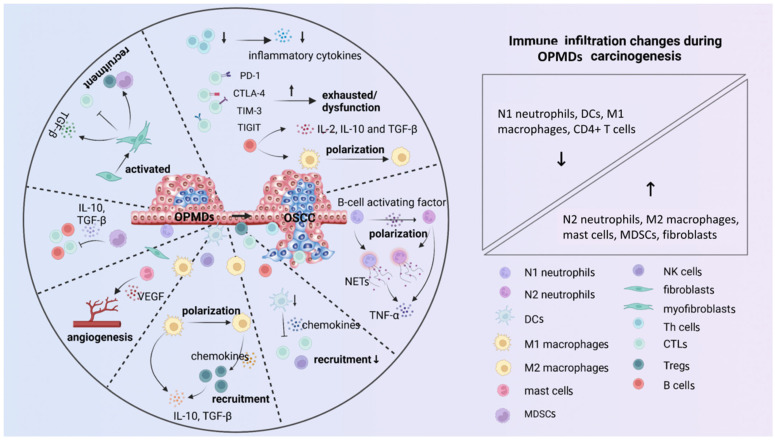
The immunosuppressive microenvironment gradually develops as OPMDs progress. The infiltration of immune cells changes with the progression of OPMDs (right panel). The immune cells convert toward inhibitory phenotypes (N2 neutrophils and M2 macrophages), produce anti-inflammatory mediators (NETs, IL-10, TGF-β, and IL-2), and recruit immunosuppressive cells (Tregs and MDSCs) that favor potentially malignant cells capable of immune evasion. Furthermore, increased angiogenesis mediated by VEGF accelerates the carcinogenesis process. Pointed arrows mean “promote” and blunt-end arrows mean “inhibit”. The upward arrows “↑” indicate “increase”, and downward arrows “↓” indicate “decrease”. NETs, neutrophil extracellular trap; TNF-α, tumor necrosis factor-α; IL-10, interleukin-10; TGF-β, transforming growth factor-β; PD-1, programmed cell death protein-1; CTLA-4, cytotoxic T-lymphocyte antigen-4; TIM-3, T cell immunoglobulin, and mucin domain-containing protein-3; TIGIT, Ig and ITIM domains; VEGF, vascular endothelial growth factor.

The immune microenvironment refers to the complex milieu associated with various cellular and non-cellular components, including immune cells, cytokines, and cell-surface molecules [14]. It mainly exerts immunosurveillance, and plays a vital role in maintaining microenvironment homeostasis [15]. However, the immune microenvironment gradually becomes suppressive as OPMDs progress and favors the immune escape (Figure 1).

### 2.1. Cellular Components

The cellular components of the immune microenvironment is mainly comprised of immune cells and fibroblasts. The former is divided into innate and adaptive immune cells, while the fibroblasts are now acknowledged as a non-classical branch of the innate immune system [16,17]. The innate immune cells exert immune effects through phagocytosis, antigen presentation, and cytokine secretion, such as interleukin-1 (IL-1), IL-6, and tumor necrosis factor-α (TNF-α) [18]. Additionally, the adaptive immune cells are involved in immunosurveillance through humoral and cellular immune responses [19]. The cell components change significantly during OPMDs carcinogenesis, from a protective to a carcinogenic response, which determines the fate of the OPMDs progression, and is an exciting issue to figure out.

Neutrophils are the first line of innate immune defense, maintaining the chronic inflammatory state by circulating cytokines [20]. Neutrophil infiltration is observed in both OLP and OLL [21]. Furthermore, increased activated neutrophils from the peripheral blood flow into the oral tissue in the presence of chronic inflammation [22]. Neutrophils are proven to be associated with the potential transformation to OSCC. The N1 to N2 phenotype conversion of neutrophils triggered by the B-cell activating factor inhibits the immune response in OLP and, thus, may favor the carcinogenesis progression [23]. N1 and N2 neutrophil phenotypes are classified depending on their functions. N1 neutrophils mainly promote immune responses, while N2 neutrophils exhibit immunosuppressive effects [20]. In addition, neutrophils can also produce neutrophil extracellular traps (NETs) to participate in the progression of OPMDs [24]. Existing evidence indicates that the role of NETs in OLP carcinogenesis is relatively complex. Jablonska et al. [25] reveal that the neutrophils isolated from OLP exhibit a strong ability to produce NETs, with the accompanying characteristic changes of components such as citrullinated histone H3 and myeloperoxidase. The citrullinated histone H3 stimulates the immune cells to generate TNF-α. TNF-α is proven to induce changes in both stroma and epithelial cells, by enhancing the invadopodia development of keratinocytes and matrix degradation [26,27,28]. However, the myeloperoxidase in neutrophils serves a vital role in killing the potentially malignant cells [29]. Hence, NETs production shows bidirectional functions, both in carcinogenic and anti-carcinogenic effects, by immune regulation and cell behavior modulation. It is not currently clear which effect is dominant.

Dendritic cells (DCs) are considered the most professional antigen-presenting cells bridging innate immunity to adaptive immunity. They play essential roles in immunosurveillance, mainly through T cell activation [30]. Compared with OSCC, more CD1a+ and CD207+ DCs infiltrate in OSF and OLK [31]. DCs are proven to inhibit the carcinogenesis of OPMDs. Anna et al. [32] found that the vaccine, which is a mix of DCs collected from femur bone marrow and premalignant tissue lysate, significantly decreases the lesion burden in 4NQO-treated mice. It can increase the total infiltration of lymphocytes and their immunosurveillance function, thus, preventing the carcinogenesis of OPMDs effectively [32].

Macrophages perform a potent immunosurveillance function, based on their ability in phagocytosis, cytokine secretion, and antigen presentation [33]. Macrophages can be typically divided into M1- and M2-polarized subtypes, according to their functions. M1 macrophages are pro-inflammatory, while M2 macrophages are anti-inflammatory [34]. Due to their indispensable roles in modulating the immune response, macrophages are essential in OPMDs carcinogenesis. The infiltration of macrophages, especially CD163+ M2 macrophages, is positively associated with the carcinogenesis of OPMDs [35]. Shigeoka et al. [36] found that OLK with high CD163+ M2 macrophage infiltration is associated with higher degrees of epithelial dysplasia. The M2 macrophages exert strong immunosuppressive and pro-carcinogenic effects, by producing anti-inflammatory cytokines, especially IL-10, and promoting the activation and function of Tregs [36]. However, Bouaoud et al. [37] observed an unexpected anti-carcinogenic effect of M2 macrophages. The high expression of the M2 macrophage gene is significantly positively correlated with oral cancer-free survival in 4NQO-treated OPMDs mice. This may be relevant, as the biological features of macrophages in oral carcinogenesis differ drastically depending on the anatomical compartment that they infiltrate [38]. Meanwhile, macrophages are extremely plastic during macrophage polarization, so they may not be completely either the M1 or M2 type, which affects the OPMDs carcinogenesis in different ways [37]. All in all, the more complex roles of macrophages require more attention and further investigation.

Mast cells are major innate and adaptive immune effector cells by potent degranulation [39]. Previous studies show that the mast cells may be involved in OPMDs carcinogenesis [40,41]. The mast cell density is statistically higher in OSCC than in OPMDs [40,41]. In addition, the mast cells promote angiogenesis through improving the expression of angiogenic factors, such as vascular endothelial growth factor (VEGF) and basic fibroblast growth factor in vascular endothelial cells [40,41]. The increased angiogenesis is a sign of carcinogenesis. That is, one of the mechanisms of mast cells promoting carcinogenesis is to promote angiogenesis, but more mechanisms remain to be studied.

Myeloid-derived suppressor cells (MDSCs) can induce strong immunosuppressive activity by expressing various cytokines and inhibiting the activity of T and NK cells [42]. MDSCs are also intimately associated with OPMDs carcinogenesis. The infiltration of CD33+ MDSCs in OSCC is significantly higher than in OPMDs. Further, MDSCs induce immunosuppression via inhibiting the activation and function of NK cells and cytotoxic T lymphocytes (CTLs), and secreting various cytokines, such as IL-10 and TGF-β [43]. The increased infiltration of MDSCs is consistent with the formation of a suppressive immune microenvironment during OPMDs carcinogenesis. These results suggest that MDSCs participate in the formation of an immunosuppressive microenvironment and the carcinogenesis of OPMDs, to a certain extent.

T cells are one kind of critical adaptive immune cell, which can be further subdivided into helper T cells (Th cells), CTLs, and regulatory T cells (Tregs) according to their immune functions. Th cells mainly secrete various cytokines, such as interferon-γ (IFN-γ), TNF-α, IL-2, and IL-4, to mediate immunity. CTLs can directly kill targeted cells via cytotoxic effect. Tregs mainly secrete cytokines such as IL-2, IL-10, and TGF-β, to exert an immunosuppressive effect [44]. Different studies have controversial conclusions about various T cell subtypes infiltrations in OPMDs and OSCC. Some studies found that CD4+ and CD8+ T cells infiltration in OSCC is lower than OLK [45], while others reach the opposite conclusions [46]. Such a large discrepancy may relate to the differences in samples and experimental conditions between different studies. Although T cells infiltration is different in different studies, the immune changes mediated by T cells is consistent. The immune response mediated by T cells is gradually suppressed as the OPMDs progress. On the one hand, the Foxp3+ Treg expression is positively correlated with the grade of OLK dysplasia [47]. On the other hand, the function of CTLs is gradually inhibited with the carcinogenesis process, and the expression of inhibitory immune checkpoints, such as programmed cell death protein-1 (PD-1), cytotoxic T-lymphocyte antigen-4 (CTLA-4), and lymphocyte-activation gene-3 (LAG-3), are up-regulated [48]. In addition, the cytokines such as IL-10 and TGF-β, secreted by T cells, also increase gradually with the progression from OLK without dysplasia, low-grade OLK, and high-grade OLK to OSCC [4]. In this case, CD4+ Th cells and CD8+ CTLs become exhausted, and the anti-carcinogenesis effect is gradually diminished. Meanwhile, the activation and function of Tregs are enhanced, leading to enforced immunosuppression.

Fibroblasts are the predominant cellular components of stroma, emerging as critical immune sentinel cells in regulating the immune response [49]. They are involved in immune regulation by activating inflammatory pathways [49]. In view of the critical role of fibroblasts in immune regulation, they may also be involved in regulating OPMDs immune microenvironment. Khalid et al. [50] found that αSMA+ myofibroblasts increase with the progression of OPMDs from mild, moderate, and severe dysplasia to eventual OSCC. Functionally, the activated fibroblasts are primarily involved in immunosuppression. They recruit MDSCs and Tregs, inhibit the function of T cells, and secrete TGF-β [17]. In addition, the fibroblasts increase the risk of OPMDs carcinogenesis, by enhancing the chance of infection by exogenous pathogenic microorganisms [51]. Previous research of our group proved that oral leukoplakia-associated fibroblasts reduce CX3CL1 secretion by inhibiting the ERK signaling pathway, thus, resulting in reduced resistance of OLK to candida albicans [51]. Further, the infection of candida albicans is recognized as a promotion factor for OLK carcinogenesis [52]. There are more relationships between fibroblasts and OPMDs to be recognized, and further carcinogenic mechanisms to be discovered.

### 2.2. Cytokines and Immune Checkpoints

The non-cellular components of the immune microenvironment mainly include cytokines and immune checkpoints, which are primarily involved in mediating cell communication and the functional state of cells. They are the main regulatory factors of immune responses, and control the proliferation, differentiation, and survival of immune cells [53]. The cytokines and immune checkpoints also exert an indispensable role in the carcinogenesis of OPMDs.

With the progression of carcinogenesis in OPMDs, the production of pro-inflammatory cytokine IL-17 decreases, and the production of anti-inflammatory cytokine TGF-β increases. It is possible that the immunosuppression induced by TGF-β plays a dominant role in carcinogenesis, rather than IL-17 [54]. In addition, cytokines also exert immunomodulatory effects by influencing the activation and function of immune cells. There is a close positive correlation between various cytokines and CD4+ and CD8+ T cells in 4NQO-treated OPMDs mice [55]. The immune checkpoints, including PD-1, CTLA-4, Ig and ITIM domains (TIGIT), LAG-3, and T cell immunoglobulin and mucin domain-containing pro-tein-3 (TIM-3), are the predominant cell-surface molecules. These cell-surface molecules significantly influence the immune response and regulate the functional status of immune cells [56]. Several results show the expression of PD-1/PD-L1 in the majority of OPMDs and OSCC samples, and their expression correlates with increased progression [57,58,59]. Blocking PD-1 or PD-L1 significantly reduces the number of oral lesions, and prevents the process of carcinogenesis in 4NQO-treated mice. It significantly increases the recruitment of CD4+, CD8+, and CTLA-4+ T cells and the expression levels of IFN-γ, STAT1, and granzyme B [60,61]. CTLA-4 is another important immune checkpoint, expressed in T cells in 4NQO-treated mice [62]. Blocking CTLA-4 also results in a significant reduction in Tregs and, thus, prevents OPMDs carcinogenesis [61,63].

Overall, both cellular and non-cellular components participate in immune regulation. They regulate the proliferation, activation, and function of immune cells by secreting various cytokines or expressing various molecules. These alterations remodel an inhibitory immune microenvironment and, thus, ultimately exert a cancer-promoting effect.

## 3. Metabolic Microenvironment

**Figure 2:**
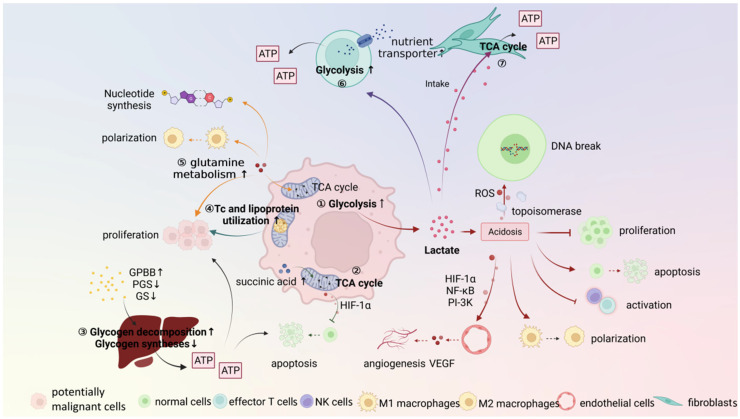
Characteristics of the metabolic microenvironment and metabolic reprogramming in carcinogenesis of OPMDs. ① The acidosis causes a series of adverse events due to increased glycolysis. ② TCA cycle also exists in potentially malignant cells and promotes cell apoptosis. ③–⑤ Metabolism of other substances, including glycogen, lipid, and protein metabolism, also participate in OPMDs carcinogenesis by providing energy and metabolic substrates. ⑥, ⑦ The metabolic reprogramming of fibroblasts and T cells is in response to the microenvironmental changes and an attempt to reverse the unfavorable conditions. Pointed arrows mean “promote” and blunt-end arrows mean “inhibit”. The upward arrows “↑” indicate “increase”, and downward arrows “↓” indicate “decrease”. ROS, reactive oxygen species; TCA cycle, tricarboxylic acid cycle; HIF-1α, hypoxia-inducible factor-1α; NF-κB, nuclear factor-κB; PI-3K, phosphatidylinositol-3 kinase; VEGF, vascular endothelial growth factor; GPBB, PGS, and GS, glycogen metabolizing enzymes; Tc, triglyceride.

The process of OPMDs carcinogenesis is due to the fact that cells constantly struggle with the surrounding environments. At the metabolic level, the potentially malignant cells constantly change their metabolic behaviors. These changes affect a wide range of cellular activities, and ultimately promote OPMDs development. The characteristics and the role of the OPMDs metabolic microenvironment is illustrated as follows (Figure 2).

### 3.1. Potentially Malignant Cells

A major feature of OPMDs carcinogenesis is the increased proliferation of potentially malignant cells. It requires an increase in metabolism to provide critical energy and substrates. However, the potentially malignant cells always proliferate away from the blood supply [64,65]. Subsequently, the high demand of metabolic activities and inadequate irrigation leads to increased anaerobic glycolysis, which leads to lactate accumulation in potentially malignant cells. At the same time, ion-exchange proteins on the cell membrane also continuously transport H+ inside the cell to the outside. These changes result in an acidic and hypoxic microenvironment. The acidic stress in a premalignant setting may increase genetic instability, including increased DNA double-strand break and the inhibition of DNA damage repair [65,66]. Moreover, the acidic microenvironment inhibits normal cell cycle progression, inhibits cell proliferation [65,67], and induces normal cell death through p53-dependent apoptotic pathways [68]. The changes in the metabolic microenvironment have a tremendous impact on cell biological behaviors: the death of normal cells increases and cells are more prone to mutation for survival. All these provide conditions for shaping a carcinogenic microenvironment.

The changes in a metabolic microenvironment, while always “carcinogenic” in general, also place selective pressure on potentially malignant cells. Only the potentially malignant cells that adapt to these conditions have a better chance of survival. The metabolic activity of potentially malignant cells increases significantly to obtain more energy and substrates. It is shown that the glycolysis is significantly enhanced in potentially malignant cells. The level of several key enzymes in glycolysis, including glucose transporter protein-1 [69,70], lactic dehydrogenase [71], and α -enolase, is found to be higher in OSCC than OPMDs. The up-regulated glycolysis promotes OPMDs carcinogenesis in multiple ways. On the one hand, the increased lactate level attenuates NK and T cell activation [72], and stimulates the polarization of macrophages to the M2 state, which leads to immunosuppression and immune evasion [73]. On the other hand, lactate also enhances the stabilization of hypoxia-inducible factor-1α, nuclear factor-κB, and phosphatidylinositol-3 kinase signaling, and induces the secretion of VEGF from endothelial cells. Thus, it participates in the proliferation and activation of potentially malignant cells and angiogenesis [74]. In conclusion, the up-regulated glycolysis allows the potentially malignant cells to acquire greater energy and substance, to better adapt to their high-cell-proliferation characteristics, and further facilitate OPMDs carcinogenesis by influencing the biological behaviors of other cells.

Other metabolic forms in OPMDs can also provide metabolic precursors for biosynthesis and energy production. The metabolic pattern of the tricarboxylic acid (TCA) cycle also exists in OPMDs, and is proven to be involved in OLP carcinogenesis. Yang et al. [75] found the accumulation of succinic acid, a key metabolite of the TCA cycle, in primary OLP keratinocytes. The accumulation of succinic acid in OLP is proven to induce apoptosis of potentially malignant cells and suppress oxidative phosphorylation, thereby reducing the risk of carcinogenesis [75]. Glycogen is another storage form of glucose. Its metabolism also affects OPMDs carcinogenesis. Compared with OPMDs, the expression of glycogenolysis enzymes, such as glycogen phosphorylase isoenzyme BB, is up-regulated in OSCC. In contrast, glycogenesis enzymes, including glycogen synthase and phosphor-glycogen synthase, are not significantly different. It suggests that the carcinogenesis of OPMDs is associated with increased glycogen synthesis. This is also consistent with increased energy metabolism in potentially malignant cells [76]. In addition, the plasma levels of total cholesterol, lipoprotein, and triglyceride in OSCC patients are significantly lower than those in OPMDs [77]. This is due to increased lipid metabolism in potentially malignant cells, leading to the constant lipid depletion in the body. The increased lipid metabolism can provide large amounts of energy and substrates for the rapid division and proliferation of potentially malignant cells [78]. Due to the increased biosynthesis of potentially malignant cells during carcinogenesis in OPMDs, the demand for nitrogen is increased. Therefore, the amino acid metabolism also plays a vital role in carcinogenesis [79]. Previous studies show that amino acid metabolism disorders may be involved in the carcinogenesis of OPMDs, by affecting the biosynthesis and metabolism of potentially malignant cells [79]. Chen et al. [80] identified several key differential metabolites, including pyruvate, glutamine, methionine, and lysine, from oral potentially malignant cells and normal human oral epithelial cells. They may contribute to the carcinogenesis of OPMDs by reprogramming the metabolism of potentially malignant cells [80]. In addition, the serum glutamine levels are also observed to increase approximately 2–26 folds in different stages of epithelial dysplasia and OSCC in DMBA-induced hamster models of oral carcinogenesis [81]. More mechanisms of amino acid in carcinogenesis of OPMDs require further investigation.

### 3.2. Stroma Cells

The metabolic activities of epithelial cells and their reprogramming of the metabolic environment play an essential role in carcinogenesis. However, the shaping of the metabolic microenvironment by stroma cells cannot be ignored. The stromal cells are influenced by the surrounding microenvironment and undergo metabolic reprogramming. It makes them better adapted to the changes in the microenvironment and attempts to reverse the unfavorable conditions.

As the most abundant component in the stroma, the metabolic activities of fibroblasts in shaping OPMDs microenvironment cannot be ignored. Our previous results suggest that the lncRNAH19/miR-675-5p/PFKFB3 signaling pathway is involved in glycolytic reprogramming of cancer-associated fibroblasts (CAFs) and promotes OSCC progression [82]. However, the metabolism of fibroblasts in OPMDs seems to play an unexpected anti-carcinogenic role [65]. Both the stromal and potentially malignant cells can process lactic acid collaboratively, to eliminate the extracellular lactic acid load and avoid large-scale microenvironment acidification. The extracellular lactic acid produced by potentially malignant cells can be taken up by fibroblasts and enters the TCA cycle [83]. Subsequently, the excess lactic acid is consumed. In other words, on the one hand, the fibroblasts can utilize the generated energy to supply their own survival and activities. On the other hand, fibroblasts can avoid the formation of an acidic microenvironment and favor their own survival. Similarly, the immune cells also attempt to survive and exert immune function by metabolic reprogramming. The aerobic glycolysis rate of T cells is up-regulated, which is consistent with the nutrient transporters and glycolysis enzymes. The increased glycolysis improves ATP availability to meet energy requirements and provide the metabolic precursors necessary for effector function and proliferation [84]. In summary, the representative metabolic microenvironment in carcinogenesis of OPMDs is the essential selective factor for both potentially malignant cells and stroma cells. The potentially malignant cells remodel the microenvironment into a hypoxic and acidic one, which is beneficial to themselves, and inhibits the growth and function of other cells. Though the other cells also undergo metabolic reprogramming and try to change the unfavorable conditions, it, ultimately, still fails to arrest OPMDs progression.

## 4. Mechanical Microenvironment

**Figure 3:**
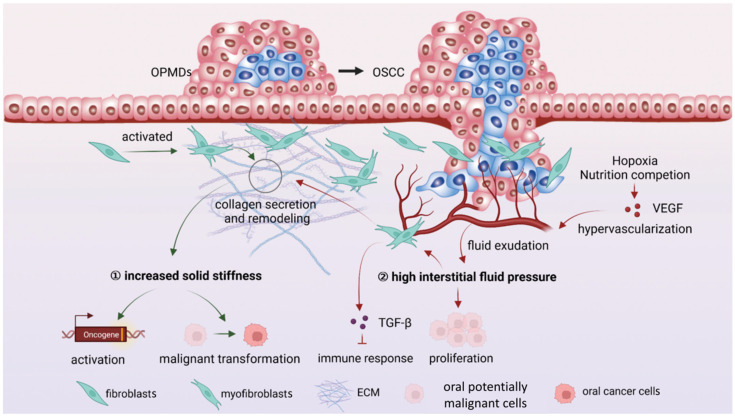
The changes and their carcinogenic roles of the mechanical microenvironment in OPMDs. ① The matrix stiffness gradually increases, promotes oncogene activation, and induces cell malignant transformation. ② The interstitial fluid pressure increases due to the fluid exudation from excessive blood vessels into the ECM and promotes carcinogenesis by inhibiting immune response and promoting cell proliferation. Pointed arrows mean “promote” and blunt-end arrows mean “inhibit”. VEGF, vascular endothelial growth factor; ECM, extracellular matrix.

The epithelial cells with carcinogenic mutations are not always sufficient to cause cancer. Although immune and metabolic microenvironments play promotive roles, the living environment of potentially malignant cells largely determines their fate. Therefore, the mechanical microenvironment, which represents the physical properties of the environment around potentially malignant cells, cannot be ignored. The process of carcinogenesis is always accompanied by increased matrix stiffness and higher interstitial fluid pressure [85]. These mechanical properties are closely related to the carcinogenesis of OPMDs (Figure 3).

The increased tissue stiffness due to ECM deposition is a classic characteristic of carcinogenesis. Compared with OSF without epithelial dysplasia, the number of αSMA+ myofibroblasts in OSF with epithelial dysplasia increases. The fibroblasts increase matrix stiffness by collagen secretion [86]. Meanwhile, Young’s moduli, which represent the rigidity of the object, are also significantly higher in OLK than OSCC [87]. The increased matrix stiffness is closely associated with carcinogenesis. When the premalignant epithelial cells adhere to the rigid collagen matrix, the oncogenic pathways and expression of oncogenes are activated [88]. In addition, the increased matrix stiffness is shown to inhibit cell senescence through the YAP/TAZ signaling pathway [89]. Also, the increased matrix stiffness is proven to enhance cell survival, proliferation, stemness, EMT, and anchorage-independent growth. These actions act synergistically with the inhibition of cell senescence to promote OSF cell carcinogenesis [90]. Overall, the increased matrix stiffness acts as a “mechanical force“ to mediate a series of carcinogenic processes, including oncogene activation, cell proliferation, migration, and senescence.

The proliferation of potentially malignant cells makes them move further away from lymphatic and blood vessels, depriving them of nutrients and oxygen. To counteract this, the potentially malignant cells release large amounts of pro-angiogenic factors, such as VEGF and basic fibroblast growth factor, into the surrounding microenvironment, promoting the rapid formation of new blood vessels. This sudden hypervascularization compresses the growing premalignant tissues. When the fluid of blood vessels leaves the vascular system and enters ECM, the overall interstitial hydrodynamic pressure increases [85]. The increased interstitial hydrodynamic pressure is proven to participate in the carcinogenesis process [91]. On the one hand, the high pressure and mechanical stretching of the cell itself can directly promote cell proliferation [91]. On the other hand, the increased fluid flow in tissue leads to more consistent changes in fibroblasts and collagen fibers, resulting in increased matrix stiffness [92]. Further, the fluid exercise stimulates fibroblasts to produce TGF-β, thereby altering the activation status of fibroblasts and inducing sustained immunosuppressive activities [93]. In conclusion, the increased matrix stiffness and interstitial fluid pressure provide another perspective to understanding OPMDs carcinogenesis through mechanical properties, and offers unprecedented possibilities for cancer prevention in the future.

## 5. Neural Microenvironment

**Figure 4:**
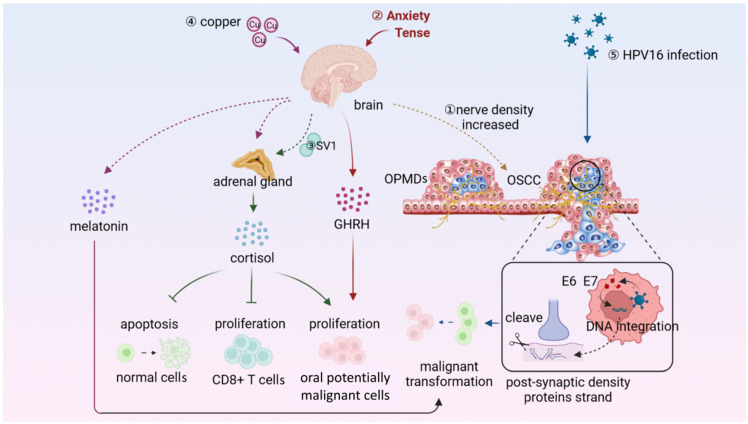
The disturbed neural microenvironment participates in the carcinogenesis of OPMDs. ① The nerve fiber density increases as OPMDs carcinogenesis progresses. ②–⑤ The external factors, including negative emotion, SV1, Cu, and HPV16 infection, significantly affect the behaviors of both potentially malignant cells and immune cells by neural regulation. The solid arrows represent direct action to the downstream components, while the dotted arrows represent that there are more intermediate multi-step reactions that are not shown. Pointed arrows mean “promote” and blunt-end arrows mean “inhibit”. GHRH, growth hormone-releasing hormone; SV1, splicing variant1; Cu, trace element copper; HPV16, human papillomavirus type 16; E6 and E7, viral proteins.

There is growing evidence that the potentially malignant cells also utilize the neural system to promote their growth and progression. Communication between nerves and potentially malignant cells mediated by neurotransmitters or neuropeptides establishes a specialized, localized microenvironment called “neural microenvironment” [94] (Figure 4).

The neural system regulation is equally important in OPMDs carcinogenesis. It is shown that the nerve density nearly doubles as carcinogenesis progresses from premalignant lesions to overt cancers [95]. Further, the disorders of neuroendocrine function are closely related to OPMDs carcinogenesis [7]. There is a higher cortisol level in OSCC patients compared with OSF patients [7]. It is reported that the disorder of cortisol secretion resulting from chronic anxiety and depression could affect the process of OPMDs carcinogenesis, to varying degrees [7]. The excessive cortisol promotes the apoptosis resistance, proliferation, and invasion of potentially malignant cells, and is closely related to inhibition of CD8+ T cell proliferation [96]. Another neuroendocrine peptide hormone, the growth hormone-releasing hormone, and its regulator, the splicing variant 1, are also demonstrated promoting the carcinogenesis of OPMDs by stimulating keratinocyte proliferation [97]. In addition, some exogenous factors can influence OPMDs progression by regulating innervation. The HPV proteins E6 and E7 integrate into host DNA and cleave the post-synaptic density protein strands after infection. Subsequently, it drives the metaplasia of host cells [98]. Also, the trace element can regulate the secretion of hormones and, thus, participate in OPMDs carcinogenesis as one kind of exogenous factor. The expression level of copper is high in OSF, and is confirmed to promote carcinogenesis. It absorbs and reduces catecholamine levels. Further, it regulates the synthesis and secretion of melatonin and other hormones [99]. In conclusion, not only the tissue innervation disorders and neuroendocrine dysfunction but also the external factors, such as virus infection and trace elements, disrupt the balance of neural microenvironment. The disturbed neural microenvironment then participates in the carcinogenesis of OPMDs by affecting the proliferation, apoptosis, and abnormal hyperplasia of potentially malignant cells.

## 6. Interaction Networks among Microenvironments

**Figure 5:**
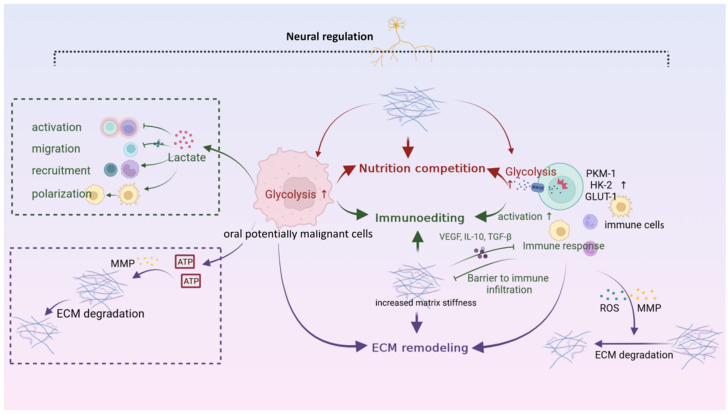
The immuno–metabolic–mechanical–neural interaction network affects carcinogenesis of OPMDs profoundly. The metabolic activities of potentially malignant cells negatively affect the immune microenvironment and remodel ECM. While the immune cells upregulate glycolysis and are activated to perform nutrition competition, immunoediting, and ECM remodeling against potentially malignant cells. Meanwhile, ECM undergoes remodeling to adapt to the changes of microenvironments. Pointed arrows mean “promote” and blunt-end arrows mean “inhibit”. The upward arrows “↑” indicate “increase”. ROS, reactive oxygen species; MMP, matrix metalloproteinase; ECM, extracellular matrix.

Each microenvironment promotes carcinogenesis in its own way. In addition, there are immuno–metabolic–mechanical–neural interaction networks, which are used to characterize the close connections between different microenvironments. Any changes in one of these microenvironments causes a series of chain reactions, leading to microenvironment perturbations and amplifying their pro-carcinogenic effects (Figure 5).

The acidic microenvironment induced by increased glycolysis of potentially malignant cells inhibits immune responses [8]. Firstly, the CD4+ T cells migration is hampered, due to chemokine receptor CXCR3 and its ligand CXCL10 [9]. Secondly, the immune surveillance is impaired, resulting in Treg recruitment, CTLs exhaustion, and the immunosuppressive phenotype of DCs [100]. Metabolism of other substances, such as amino acids and lipids, also inhibit effector T cells activities and decrease effector T cells/Tregs ratios, leading to immunosuppression [101]. In contrast, the immune cells undergo metabolic reprogramming to adapt to and change the metabolic microenvironment. The transcriptional activity of MYC and HIF-1 in T cells is increased, and critical glycolysis enzymes such as pyruvate kinase1, hexokinase, and glucose transporter1 are up-regulated, providing metabolic precursors necessary for effector factors function and proliferation [102]. However, the metabolic adaptive behaviors of immune cells are inhibited as OPMDs progress. Worse, the metabolic reprogramming skews into the pro-carcinogenic direction. For example, M2 macrophages up-regulate mitochondrial respiration and metabolize amino acids differently from inflammatory macrophages. They express high levels of arginase 1, consume arginine, and produce highly immunosuppressive polyamines instead [103].

The alterations in the metabolic microenvironment allow potentially malignant cells to dynamically adjust energy production and bioenergy, in response to fluctuating energy requirements, and better adapt to and remodel the microenvironment [104]. The metabolic activities of OPMDs potentially malignant cells can also significantly remodel the ECM. The glycolysis of potentially malignant cells increases, and the energy generated could be used to transport matrix metalloproteinase (MMP) to the front of invasion and degrade the ECM [105]. In addition, the ROS produced by neutrophils and monocytes could degrade ECM components, mainly type I collagen, and stimulate the production of MMP [106]. Meanwhile, the change in the mechanical microenvironment leads to differences in the cell space environment, which profoundly affects cell metabolism. The matrix stiffness increases glycolysis and glutamine consumption of CAFs and OSCC cells. In particular, the activation of the YAP/TAZ signaling pathway promotes glycolytic enzyme expression, glutamine uptake, and transformation to glutamate [10]. Additionally, the matrix stiffness/YAP/TAZ pathway is shown to mediate OSF carcinogenesis through the senescence escape pathway. Thus, the YAP/TAZ signaling pathway affects both metabolic and mechanical microenvironments simultaneously, and promotes carcinogenesis synergistically [107].

The ECM is highly ordered and closely arranged around the lesion, preventing immune cells from entering and forming a natural barrier to immune infiltration. Meanwhile, changes in the structure and composition of the ECM under disease conditions provide physical and biochemical cues to immune cells that influence their activation state [11]. The ECM alters the ratio of effector T cells to Tregs by secreting anti-inflammatory cytokines and growth factors such as VEGF, IL-10, and TGF-β [108]. In contrast, the immune cells also participate in the decomposition, synthesis, and remodeling of the ECM [109]. For instance, the infiltrated M2 macrophages induce ECM hydrolysis, collagen endocytosis, and lysosome degradation by up-regulating MMP [110]. Additionally, immune cells could induce ECM remodeling by activating fibroblasts. Persistent inflammatory stimulation and TGF-β secreted by immune cells result in the overactivation of myofibroblasts, leading to adverse ECM deposition [111]. The ECM can generally regulate the immune response, while the infiltrated immune cells tend to remodel the mechanical microenvironment. However, it ultimately tilts the balance in the pro-carcinogenic direction. 

The neural microenvironment acts as a “command center” that regulates the immune response, metabolic activity, and ECM remodeling through synaptic connections or the secretion of various neurotransmitters. The neural system influences the immune response by regulating the metabolic behaviors of immune cells. For example, norepinephrine reduces glycolysis and inhibits the activation of CD8+ T cells through a pathway induced by β2-adrenergic receptors [112]. In addition, the nervous system alters endothelial cell metabolism, and acts as a “vascular metabolic switch” to regulate the initiation and pattern of angiogenesis. In a mouse model of prostate cancer, sympathetic nerve excision regulates the progression of premalignant cancer from low-grade to the high-grade malignant stage by up-regulating cytochrome C oxidase assembly factor, turning endothelial cell metabolism from glycolysis to oxidative phosphorylation, and inhibiting angiogenesis [113]. The neural microenvironment is also an essential regulatory component of ECM remodeling. In a pancreatic ductal carcinoma mouse model, stress-induced adrenergic signal enhancement increases MMP expression in the stromal compartment by more than 100 times [114]. Most of the studies about the interactions between neural microenvironment and other microenvironments are in tumors. In view of the strong regulatory role of neural microenvironment, it is believed that the interaction between OPMDs and other microenvironments also exists in OPMDs, and the carcinogenic mechanisms deserve further explorations.

## 7. Microenvironment-Based Targeting Strategies

Significant breakthroughs in understanding the microenvironmental changes that drive carcinogenesis of OPMDs and the concept of targeting microenvironments, offer unprecedented possibilities for cancer prevention. We summarize current clinical trials of therapeutics for OPMDs carcinogenesis prevention in Table 1.

The significant changes in the immune microenvironment during the carcinogenesis of OPMDs make immunotherapy one of the most promising strategies for cancer prevention. Vaccines are the most commonly applied immune-based interventions, which induce an adaptive immune response by promoting antigen presentation [115]. Several studies show the sharing of tumor antigens between OPMDs and OSCC. For example, similar tumor antigens exist between the 4NQO-treated OPMDs mice and OSCC developed from these lesions [116]. DC vaccine, a mix of dendritic cells and premalignant tissue, stimulates the immune reactivity in vitro and in mouse studies by increasing the expression of CD8+ T cells and IFN-γ and, thus, decreasing the occurrence of OSCC [117]. Therefore, identifying and targeting up-regulated specific antigens is promising in inhibiting OPMDs carcinogenesis. Furthermore, targeting high-expressed immune checkpoints to enhance immune response is effective. Blocking the PD-1/PD-L1 pathway is demonstrated to prevent the progression of carcinogen-induced OPMDs in mice models [118]. At present, many immune checkpoint inhibitors have entered clinical trials. A phase II clinical trial that used aviluzumab to treat OPMDs shows significant effects [119]. Non-specific immunomodulators can either activate or suppress the immune system in a general non-specific way, and have the potential to be tested for cancer prevention [120]. It is demonstrated that prostaglandin biosynthesis inhibitors stimulate levels of IFN-γ-expressing CD8+ cells locally within regional lymph nodes and distally in the spleen, and decrease OPMDs carcinogenesis in mice models [121]. Some old drugs–new uses also have identified sound clinical effects, and potent immune activity. Metformin could prevent the development of OSCC by significantly reducing the size and number of carcinogen-induced oral tumoral lesions, and preventing their spontaneous malignant transformation [122].

The characteristic reprogramming of the metabolic microenvironment occurs during carcinogenesis of OPMDs, and potentially malignant cells develop alternative compensatory metabolic changes. In light of this, systemic manipulations to restore the metabolic state of potentially malignant cells to a normal cell state, thereby decreasing the occurrence of OSCC, are desired. The targeted metabolic microenvironment mainly focuses on blocking glucose metabolism. There is a progressive glycolytic perturbation, which is attenuated by salvianolic acid B treatment, and decreases incidences of OSCC in dimethylbutane-acid-induced OPMDs hamsters. Further research found that Sal-B treatment down-regulates the PI3K and HIF-1α signaling pathways to inhibit aerobic glycolysis, thus, inducing premalignant cell death and reducing OSCC formation [123]. In addition, glutamine metabolism is up-regulated during the carcinogenesis of OPMDs. Glutaminase1 selective inhibitor curtails glutamine consumption, and inhibits HNSCC cell growth by inducing apoptosis and cell cycle arrest. Meanwhile, metformin could enhance the antitumor effect by inhibiting the glucose utilization of potentially malignant cells [124].

Targeting the mechanical microenvironment is a promising strategy to prevent OSCC by reversing ECM remodeling. Metformin effectively reduces ECM components such as collagen type VI, MMP2, and MMP9 [125]. It is proven to reduce the size and number of 4NQO-treated OPMDs mice [126]. The microenvironment-based targeting strategies seem to revolutionize the future of cancer prevention, and offer unprecedented possibilities, which require more studies.

## 8. Conclusions

Microenvironmental perturbations are primary in the carcinogenesis of OPMDs. The potentially malignant cells shape the immunosuppressive, hypoxic, acidic, and stiff microenvironment, which ultimately promotes carcinogenesis. In addition, the immuno–metabolic–mechanical–neural interaction networks further amplify these pro-carcinogenesis effects. The awareness and knowledge of the characteristics and interactions of the microenvironments make the microenvironment-based monitoring and targeting strategies for the prevention of OPMDs carcinogenesis provides more perspective.

Monitoring the risk of OPMDs carcinogenesis is of critical significance in OSCC prevention. We developed a personalized computing model for predicting the risk of OPMDs carcinogenesis, and established a Chinese–English bilingual website (opmd-risk.com, accessed in 1 July 2022) to promote and apply the prediction system [127]. In the near future, we will consider integrating the microenvironment characteristics into the prediction system. In addition to monitoring the risk of OPMDs carcinogenesis, it is of equal importance to take measures to prevent the carcinogenesis of OPMDs. Considering the close interactions between microenvironments, we propose that targeting multiple microenvironments with common targets may achieve synergistic efficacy in cancer prevention. This is probably attributed to the previously described interaction network among multiple microenvironments. For example, when the activity and function of immune cells are enhanced, the immune response is augmented. Meanwhile, the activated immune cells perform in nutrient competition with potentially malignant cells, which can correct the metabolic microenvironment to some extent. In addition, ROS and MMP produced by immune cells can promote ECM degradation, and alleviate the microenvironment stiffness. Further, inspired by the sequential target perturbation strategy, we proposed a similar strategy in cancer cells and CAFs. That is, CAFs are first targeted to block the protumor niche, and then cancer cells are treated to achieve anticancer effects [49]. Here, we further present that, on the basis of targeting microenvironments, the combination of targeting potentially malignant cells and microenvironments may have a “1 + 1 > 2” effect in the context of OPMDs carcinogenesis prevention. Future studies are needed to verify these hypotheses.

Current prevention strategies for carcinogenesis of OPMDs mainly focus on chemoprevention, surgical resection, and photodynamic therapy. Drugs such as retinoids, vitamin A, and/or β-carotene can achieve clinical efficacy to some extent. However, when the carcinogenesis is ongoing, this traditional chemoprevention has a limited clinical response. Surgical intervention can resect the potentially malignant tissue. However, it cannot eliminate carcinogenesis because of the existence of cancer-initiating microenvironments. The second surgery is intractable and not always acceptable for patients when the lesion relapses. Photodynamic therapy attracted interest from physicians; however, patients need to be treated multiple times in the clinic, and the treatment is time-consuming. Recently, a growing number of studies turned their attention to developing new chemoprevention [128,129,130]. The multi-dimensional changes in microenvironments of OPMDs will provide new directions for promising chemoprevention targets. However, there is a lack of specific biomarkers for oral carcinogenesis. Furthermore, the databases for the molecular targets of each microenvironment in individual OPMDs are still not well-established. More studies are required to set up the databases for common target prediction. Additionally, the existing animal models of oral carcinogenesis are mainly induced by 4NQO, which cannot adequately mimic the characteristics of individual OPMDs. These are the major roadblocks to translate experimental research into clinical practice. In conclusion, there are more microenvironmental perturbations to be discovered, ways of microenvironment interactions to be explored, and common target of microenvironments to be confirmed. More ways or models for carcinogenesis prediction also merit greater attention.

## Figures and Tables

**Table 1:** Recently completed and ongoing chemoprevention of OPMDs carcinogenesis (data collected from https://clinicaltrials.gov, accessed on 3 June 2022).

Drug Type	Drug	OPMDs Type	Result	Status	NCT Number	Last Update Date
mAb	Avelumab	OPMDs with dysplasia	No results posted.	Phase II; recruiting	NCT04504552	2020
	Sintilimab	High-risk OPMDs	No results posted.	Phase II; not yet recruiting	NCT04065737	2019
	Nivolumab	OVH	No results posted.	Phase II; active, not recruiting	NCT03692325	2021
	Pembrolizumab	OLK; OEL and OVH	No results posted.	Phase II; recruiting	NCT03603223	2021
	Celecoxib	OLK	Could not prevent the disease progression.	Phase II; completed	NCT00101335	2013
	Celecoxib	OPMDs with dysplasia	Prevented oral cancer from forming.	Phase II; completed	NCT00014404	2013
	Celecoxib	OLK with dysplasia	No results posted.	Phase II; completed	NCT00052611	2018
	Celecoxib	OPMDs	No results posted.	Phase II; completed	NCT00036283	2008
Vaccine	Hespecta vaccination	HPV16-positive OPMDs	No results posted.	Phase I; completed	NCT02821494	2021
Plant extract and mAb	Green tea polyphenon E and erlotinib	OPMDs with dysplasia	No results posted.	Phase I; completed	NCT01116336	2018
Plant extract	Bowman–Birk inhibitor Concentrate	OLK	Prevented oral cancer from forming.	Phase II; completed	NCT00330382	2014
Plant	Green tea	OLK	No results posted.	Phase II; terminated	NCT00176566	2009
Hypoglycemic drug	Metformin hydrochloride	OLK and OEL with dysplasia	Prevented oral cancer from forming.	Phase II; completed	NCT02581137	2021
	Pioglitazone hydrochloride	OLK	No significant effect.	Phase II; terminated	NCT00951379	2016
	Rosiglitazone	OLK and OEL	Prevented oral cancer from forming.	Phase II; completed	NCT00369174	2013
	Pioglitazone hydrochloride	OLK with dysplasia	Prevented oral cancer from forming.	Phase II; completed	NCT00099021	2019
TKI	Vandetanib	OPMDs with dysplasia	Prevented oral cancer from forming.	Phase II; completed	NCT01414426	2021
	EGFR and COX-2 inhibitor	OPMDs	No significant effect.	Phase I/II; completed	NCT00314262	2014
Analgesic–antipyretic	Aspirin mouthwash	OLK	Prevented oral cancer from forming.	Phase I; completed	NCT01238185	2013
	Sulindac	OLK and OEL	No results posted.	Not applicable; has results	NCT00299195	2020
Vitamin A derivative	SBS-101/Isotretinoin oral-adhesive film	OLK and OEL	No results posted.	Phase I; withdrawn	NCT03939364	2021

OPMDs, oral premalignant diseases; OLP, oral lichen planus; OLK, oral leukoplakia; OEL, oral erythroleukoplakia; OVH, oral verrucous hyperplasia; mAb, monoclonal antibody; TKI, tyrosine kinase inhibitor, EGFR, epidermal growth factor receptor; COX-2, cyclooxygenase-2.

## Abbreviations

OPMDs	Oral potentially malignant disorders
OSCC	Oral squamous cell carcinoma
OLK	Oral leukoplakia
OLP	Oral lichen planus
OSF	Oral submucous fibrosis
OLL	Oral lichenoid lesions
OEL	Oral erythroleukoplakia
ECM	Extracellular matrix
IL	Interleukin
TNF-α	Tumor necrosis factor-α
NETs	Neutrophil extracellular traps
DCs	Dendritic cells
4NQO	4-nitroquinoline-oxide
MDSCs	Myeloid-derived suppressor cells
Th cells	Helper T cells
CTLs	Cytotoxic T lymphocytes
IFN-γ	Interferon-γ
Tregs	Regulatory T cells
PD-1	Programmed cell death protein-1
CTLA-4	Cytotoxic T-lymphocyte antigen-4
TIGIT	Ig and ITIM domains
LAG-3	Lymphocyte-activation gene-3
TIM-3	T cell immunoglobulin and mucin domain-containing protein-3
VEGF	Vascular endothelial growth factor
TCA	Tricarboxylic acid
CAFs	Cancer-associated fibroblasts
MMP	Matrix metalloproteinase
